# Horizontal Transmission of Cytosolic Sup35 Prions by Extracellular Vesicles

**DOI:** 10.1128/mBio.00915-16

**Published:** 2016-07-12

**Authors:** Shu Liu, André Hossinger, Julia P. Hofmann, Philip Denner, Ina M. Vorberg

**Affiliations:** a German Center for Neurodegenerative Diseases (DZNE e.V.), Bonn, Germany; b Rheinische Friedrich-Wilhelms-Universität Bonn, Bonn, Germany

## Abstract

Prions are infectious protein particles that replicate by templating their aggregated state onto soluble protein of the same type. Originally identified as the causative agent of transmissible spongiform encephalopathies, prions in yeast (*Saccharomyces cerevisiae*) are epigenetic elements of inheritance that induce phenotypic changes of their host cells. The prototype yeast prion is the translation termination factor Sup35. Prions composed of Sup35 or its modular prion domain NM are heritable and are transmitted vertically to progeny or horizontally during mating. Interestingly, in mammalian cells, protein aggregates derived from yeast Sup35 NM behave as true infectious entities that employ dissemination strategies similar to those of mammalian prions. While transmission is most efficient when cells are in direct contact, we demonstrate here that cytosolic Sup35 NM prions are also released into the extracellular space in association with nanometer-sized membrane vesicles. Importantly, extracellular vesicles are biologically active and are taken up by recipient cells, where they induce self-sustained Sup35 NM protein aggregation. Thus, in mammalian cells, extracellular vesicles can serve as dissemination vehicles for protein-based epigenetic information transfer.

## IMPORTANCE

Prions are proteinaceous infectious particles that propagate by templating their quaternary structure onto nascent proteins of the same kind. Prions in yeast act as heritable epigenetic elements that can alter the phenotype when transmitted to daughter cells or during mating. Prion activity is conferred by so-called prion domains often enriched in glutamine and asparagine residues. Interestingly, many mammalian proteins also contain domains with compositional similarity to yeast prion domains. We have recently provided a proof-of-principle demonstration that a yeast prion domain also retains its prion activity in mammalian cells. We demonstrate here that cytosolic prions composed of a yeast prion domain are also packaged into extracellular vesicles that transmit the prion phenotype to bystander cells. Thus, proteins with prion-like domains can behave as proteinaceous information molecules that exploit the cellular vesicle trafficking machinery for intercellular long-distance dissemination.

## INTRODUCTION

Prions are self-perpetuating proteinaceous elements that encipher phenotypic information in the absence of coding nucleic acid. In 1982, Prusiner coined the term prion (proteinaceous infectious particle) to describe the unconventional nature of pathogens causing transmissible spongiform encephalopathies (TSEs) in humans and other mammals (1). TSEs are devastating neurological disorders that can be transmitted within and often between species. The main component of the infectious particle is the host-encoded prion protein, PrP^C^, a glycosylphosphatidylinositol-anchored cell surface protein of ill-defined function. The aberrant folding and aggregation of PrP^C^ into the disease-specific isoform PrP^Sc^ confer infectious properties to the protein polymer that propagates by templating its conformation onto PrP^C^. PrP^Sc^ accumulates as highly ordered aggregates with a cross-beta structure, so-called amyloid (2). While only a few prions, such as scrapie of sheep and goats and chronic wasting disease in cervids, are contagious and transmitted naturally between individuals, prions haven been experimentally or accidentally transmitted by different routes (3). Both the lymphoreticular and peripheral nervous systems appear to be involved in prion invasion of the central nervous system (4). *In vitro* and *in vivo* evidence suggests that mammalian prions exploit cell-cell contacts such as tunneling nanotubes or secreted vesicles for intercellular transmission (5–7).

Remarkably, proteins that can adopt infectious amyloid conformations are widespread in lower eukaryotes (8). Yeast (*Saccharomyces cerevisiae*) prions constitute epigenetic elements of inheritance that are associated with heritable phenotypes that can be harmful or beneficial, depending on genetics and the environment (8–10). At least nine *bona fide* fungal prions have been identified (11). Mammalian and fungal prion proteins are unrelated in amino acid sequence, yet they replicate by the same mechanism of seeded polymerization (12). Most of the yeast prions identified contain prion domains with low complexity enriched in polar amino acids (glutamine, asparagine, and tyrosine) and glycine (13–15). Elegant shuffling experiments have revealed that compositional bias rather than the primary sequence is the determinant of prion propensity (16). Adoption of the “prion state” is a rare but reversible event that can be elicited by environmental stressors (17). Prion formation most often leads to a loss of function of the respective protein, giving rise to a variety of heritable metabolic phenotypes. The translation termination factor Sup35 is the most-studied yeast prion protein (18, 19). Sequestration of Sup35 into prion aggregates results in translational readthrough and changes the metabolic phenotype of the cell. Prion domain N of Sup35 is modular and is responsible for prion formation (20), while the charged middle domain M increases the solubility of the protein in its nonprion state and is involved in prion maintenance in yeast (21). The translation termination activity of the protein is conferred by the carboxy-terminal domain of the protein, which is dispensable for prion formation (22). Yeast prions are faithfully inherited by daughter cells and horizontally transmitted during mating. Natural, nonsexual transmission of prions in lower eukaryotes has not been observed so far. However, Sup35 prions have recently been found packaged into extracellular vesicles of yeast, suggesting that secreted vesicles could serve as vehicles for the intercellular dissemination of protein-based elements of inheritance, at least in yeast (23).

Domains with amino acid composition comparable to that of yeast prion domains are also present in many mammalian proteins. Approximately 1% of the human proteome contains low-complexity domains enriched in asparagine and glutamine residues with compositional similarity to yeast prion domains (14). Many of those human proteins form functional RNA-protein complexes, and their prion-like domains are critical for the rapid self-assembly of these complexes under stressful conditions (24). Importantly, several human proteins with prion-like domains have also been associated with neurodegenerative diseases, suggesting that aberrant aggregation could also cause disease (24).

To understand if proteins with domains similar to yeast prion domains can form *bona fide* prions in mammalian cells, we have recently established a cell culture model that is based on the expression of the prion domain of Sup35 in mouse neuroblastoma cells (25). The NM domain of Sup35 shows no sequence homology with mammalian proteins and thus allows us to study prion formation without the adverse effects of any loss of function. Cytosolically expressed NM is nontoxic and nonaggregated in mouse neuroblastoma cells (25). However, exogenous NM fibrils efficiently induce self-sustained NM aggregates that are vertically transmitted to progeny. Importantly, NM prions in mammalian cells exhibit infectious properties similar to those of mammalian prions and are horizontally transmitted to bystander cells (25, 26). Cell-to-cell contact appears to be most efficient for transmitting the prion phenotype to recipient cells (26, 27). In this study, we demonstrate that a fraction of prion infectivity is also secreted in association with extracellular vesicles. NM aggregates present in exosomal fractions are biologically active and induce heritable prion phenotypes in recipient cells. Thus, mammalian cells can package protein assemblies with yeast prion domains into secreted vesicles that transmit the aggregation state to bystander cells. In light of the high number of mammalian proteins harboring low-complexity domains with compositional similarity to those of yeast prions, it is tempting to speculate that dissemination of prion-like protein assemblies could play a more general role in cell-cell communication.

## RESULTS

### Release of infectious NM-HA into the cell culture supernatant.

We have previously shown that Sup35 NM prions can be efficiently induced by coculture of mouse N2a donor cells harboring hemagglutinin (HA)-tagged NM prions (NM-HA^agg^) with recipient cells expressing soluble NM-green fluorescent protein (NM-GFP^sol^) (26). Induction depended on the transmission of NM-HA^agg^ seeds from donor to bystander recipient cells and was most efficient when cells were cultured in close proximity, strongly suggesting that direct cellular contact is the most effective route of cytosolic prion dissemination. Interestingly, residual inducing activity was detected in conditioned medium (26). Thus, at least some infectivity was also released into the cell culture supernatant.

**FIG 1:**
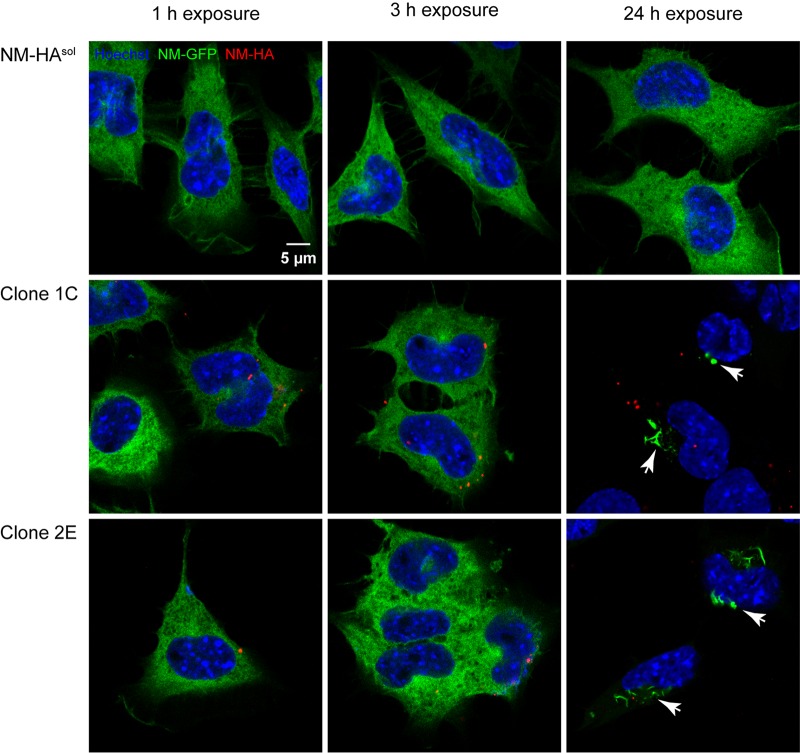
Murine N2a cells harboring cytosolic Sup35 NM-HA aggregates secrete infectious NM prions. Shown are confocal images of recipient N2a NM-GFP cells (green) exposed to the 100,000 × *g* pelleted fractions derived from conditioned medium of NM-HA aggregate-producing cell clones 2E and 1C or control donor cells expressing soluble NM-HA. Recipient cells were fixed 1, 3, and 24 h posttreatment with pellet fractions. NM-GFP aggregate induction was observed after 24 h with pellet fractions derived from media of both clones but not from donor cells expressing NM-HA^sol^. NM-HA was stained with anti-HA antibody (red), and nuclei were counterstained with Hoechst (blue). NM-GFP aggregates are indicated by arrows. For images showing cells with NM-GFP aggregates, the microscopy setting had to be adjusted to prevent the overexposure of highly fluorescent aggregates. Note that no colocalization of internalized NM-HA seeds and NM-GFP aggregates was observed. Scale bar, 5 µm.

To characterize cytosolic NM prion induction by conditioned medium in more detail, we made use of two donor clones, 2E and 1C, that were derived from a bulk population of N2a cells stably expressing NM-HA (Fig. 1A) (25). Because of previous exposure to amyloid fibrils produced by using recombinant NM protein, these cells continuously produce NM-HA^agg^ with prion properties. Individual clones that chronically produce phenotypically diverse types of NM-HA^agg^ were subsequently isolated from this bulk population (25). To concentrate potentially released NM-HA, conditioned media of control N2a cells expressing soluble NM-HA (NM-HA^sol^) and the two NM-HA^agg^ clones were subjected to high-speed centrifugation. Pellet fractions were dissolved in phosphate-buffered saline (PBS) and subsequently added to a recipient N2a cell population expressing GFP-tagged soluble NM (NM-GFP^sol^) (Fig. 1). Induction of NM-GFP aggregation was monitored up to 24 h postexposure by immunofluorescence analysis. NM-HA was observed associated with recipient cells within 1 h postexposure to pellet fractions derived from medium of NM-HA^agg^ donor clones (Fig. 1). After 24 h, recipient cells with NM-GFP aggregates were identified. Some recipient cells also contained NM-HA puncta, strongly suggesting that NM-HA secreted into the medium was taken up. Of note, in line with aggregate induction by direct cell contact (26), a large amount of NM-GFP expressed by recipient cells was sequestered into aggregates. These results argue that NM-HA released by the donor clones was infectious and induced aggregation of NM-GFP in recipient cells.

**FIG 2:**
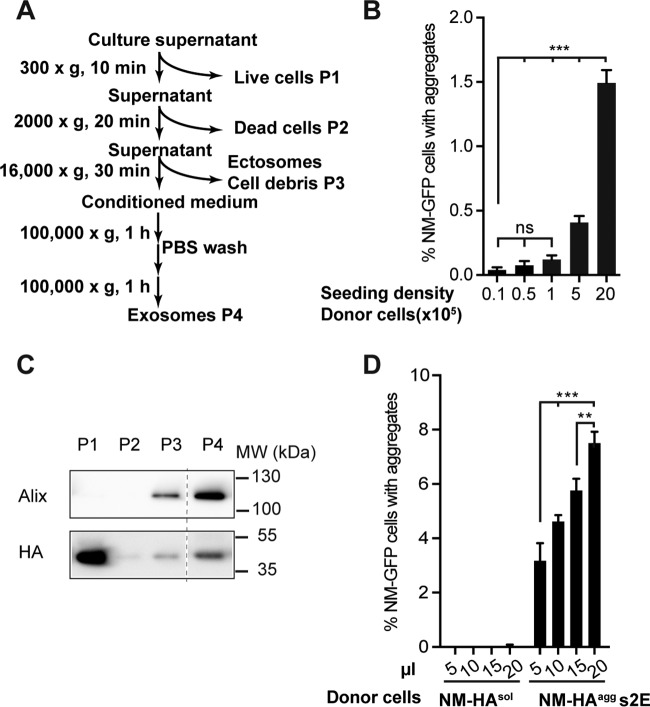
Infectious NM prions are associated with the exosomal fraction. (A) Differential centrifugation protocol to enrich for exosomes. Donor cells were grown in exosome-depleted medium. (B) Cell-based aggregate induction assay. Conditioned medium derived from NM-HA^agg^ clone s2E plated at different densities induced NM-GFP aggregation in recipient cells in a dose-dependent manner. Shown is the mean ± SD (*n* = 3). Statistical analysis was performed by one-way ANOVA. ***, *P* < 0.001. (C) Western blot analysis of pellet fractions P1 to P4 isolated from conditioned medium of s2E cells according to the scheme in panel A. Alix served as a marker protein for exosomes. Anti-HA antibody was used to detect NM-HA. Additional lanes were excised for presentation purposes (dotted line). (D) Cell-based aggregate induction assay using the exosome-enriched P4 fraction isolated from the media of NM-HA^sol^ and NM-HA^agg^ s2E cells. The pellet was dissolved in PBS, and 5 to 20 µl was added to recipient NM-GFP^sol^ cells. The number of recipient cells with induced aggregates was determined 16 h postexposure. The results shown are means ± SD (*n* = 4). **, *P* < 0.01; ***, *P* < 0.001; one-way ANOVA.

Mammalian prions *in vivo* and *in vitro* can be released in association with exosomes (5, 6). Nanometer-sized membranous vesicles such as exosomes, microvesicles, and other extracellular vesicles mediate intercellular communication by shuttling complex bioactive cargo such as lipids, nucleic acids, or proteins between cells (28). While microvesicles are produced through outward budding from the cell membrane, exosomes are released into the extracellular space upon the fusion of multivesicular bodies with the plasma membrane. To quantitatively test the aggregate induction efficiency of extracellular vesicle fractions, the aggregate induction assay was adapted to automated high-throughput confocal microscopy. An image analysis routine was developed to determine the percentage of recipient cells with induced NM-GFP aggregates (see Fig. S1 in the supplemental material). To increase the aggregate induction efficiency of conditioned medium, subclone s2E of N2a NM-HA^agg^ 2E that produces highly infectious conditioned medium was used. Conditioned medium derived from s2E (Fig. 2A) successfully induced NM-GFP aggregation in recipient cells, suggesting that donor cells released infectious NM prions into the cell culture medium (Fig. 2B). Induction was most efficient when conditioned medium was derived from high-density donor populations (Fig. 2B). Cell debris, microvesicles, and exosomes were separated by differential centrifugation (Fig. 2A) (29), and the different pellet fractions isolated from conditioned medium were tested for the presence of the exosomal marker protein Alix and NM-HA. Western blot analysis confirmed that the differential centrifugation successfully isolated exosomal fraction P4. Importantly, exosomal fraction P4 contained substantial amounts of NM-HA (Fig. 2C) and exhibited strong aggregate-inducing activity when added to recipient cells (Fig. 2D). No aggregate-inducing activity was associated with an exosomal fraction derived from control donor cells expressing NM-HA^sol^ (Fig. 2D). The earliest aggregate induction was observed by time-lapse microscopy 3 or 4 h after treatment with the exosomal fraction (see Movie S1 in the supplemental material). Further culture of the exosome-treated recipient cells over several passages demonstrated that induced NM-GFP aggregates were faithfully inherited by progeny cells and thus exhibit characteristic prion properties (data not shown).

### NM-HA and seeding activity are associated with highly purified exosome fractions.

**FIG 3:**
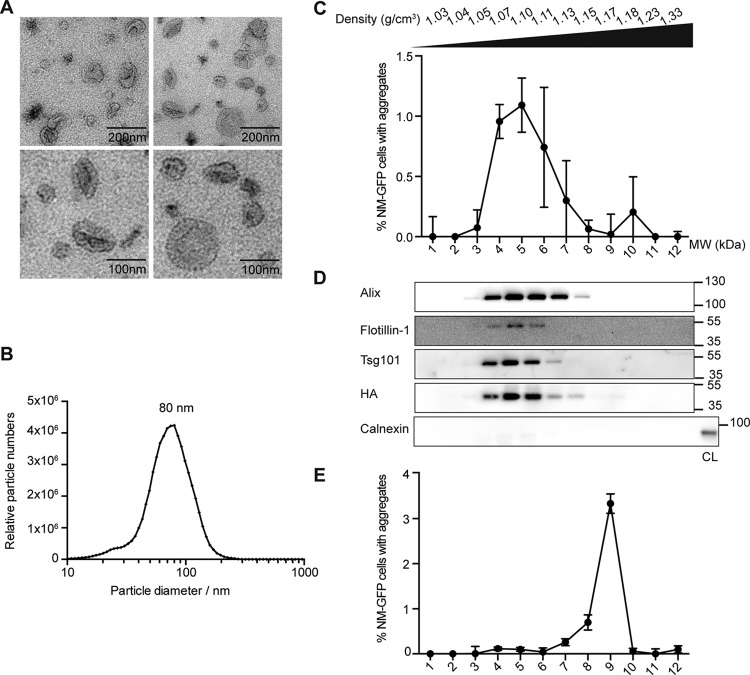
NM-HA and prion infectivity cofractionate with exosomes. (A) Transmission EM of the P4 fraction from donor clone s2E reveals the typical exosomal shape and dimension. Scale bars: top, 200 nm; bottom, 100 nm. (B) Size distribution of vesicles in the P4 fraction from media of donor clone s2E. (C) Fractionation of the exosome-enriched P4 fraction from medium of clone s2E by OptiPrep gradient centrifugation. Twelve fractions were collected and analyzed for aggregate-inducing activity in recipient NM-GFP^sol^ cells (*n* = 3). (D) OptiPrep density gradient fractions used in panel C were subjected to Western blot analysis to test for the distribution of NM-HA and exosomal marker proteins Alix, Tsg101, and Flotillin-1. Calnexin served as a marker protein for the endoplasmic reticulum. s2E cell lysate (CL) was loaded as a control for calnexin detection. Note that none of the OptiPrep fractions contained calnexin, excluding organelle contamination. Immunoblotting with anti-HA antibodies revealed that NM-HA cofractionated with exosomal markers. (E) Fractionation of *in vitro*-formed NM fibrils by OptiPrep density gradient centrifugation. The different fractions were analyzed for aggregate-inducing activity in recipient NM-GFP^sol^ cells. The highest induction rates were observed for fraction 9. The results shown are means ± SD (*n* = 3).

The foregoing experiments demonstrated that high seeding activity was associated with the exosome-enriched pellet isolated from the conditioned medium of aggregate-bearing cells. Transmission electron microscopy (EM) of the P4 fraction derived from the medium of donor clone s2E verified the presence of a heterogeneous population of small, cup-shaped vesicles that had a diameter of 30 to 120 nm, thus falling within the size range of exosomes (Fig. 3A). This size distribution was also confirmed by nanoparticle tracking analysis, which showed a peak value of 80 nm (Fig. 3B).

To further purify exosomal fractions, we combined differential centrifugation with OptiPrep density gradient (29, 30). Twelve OptiPrep fractions were collected and tested for aggregate-inducing activity (Fig. 3C). The strongest aggregate-inducing activity was associated with fractions 4 to 7, while minor inducing activity was also observed with fraction 10. The majority of exosomal markers Alix, Tsg101, and Flotillin-1 was present in fractions 4 to 7 with a density of 1.07 to 1.13 g/cm^3^ (Fig. 3D), which falls in the range of that reported for exosomes isolated from N2a cells (31). The endoplasmic reticulum-resident protein calnexin, as a marker of contaminating intracellular vesicles, was not present in any of the OptiPrep fractions, demonstrating the purity of the preparation of the exosome-enriched P4 pellet. Importantly, in fractions containing exosomal marker proteins, NM-HA was also highly abundant. No exosomal markers were found in fraction 10, which contained a very faint NM-HA signal, suggesting that seeding activity in this fraction was likely due to free NM-HA aggregates (Fig. 3C and D). Importantly, seeding activity of control OptiPrep gradient fractions containing recombinant NM fibrils was associated mainly with fraction 9 (density, 1.17 g/cm^3^) (Fig. 3E). Moreover, OptiPrep gradient fractions prepared with insoluble proteins derived from s2E cell lysates revealed seeding activity in the majority of the fractions. Seeding activity correlated with the presence of NM-HA but not with that of vesicle markers Alix and Flotillin-1 (see Fig. S2 in the supplemental material). The finding that the aggregate-inducing activity of the individual OptiPrep fractions of the exosome-enriched pellet differed from that of recombinant fibrils and s2E cell lysate fractionated by the same procedure suggests that the majority of NM-HA is directly associated with exosomes and is not freely released.

### Intact exosomes are required for efficient aggregate induction.

**FIG 4:**
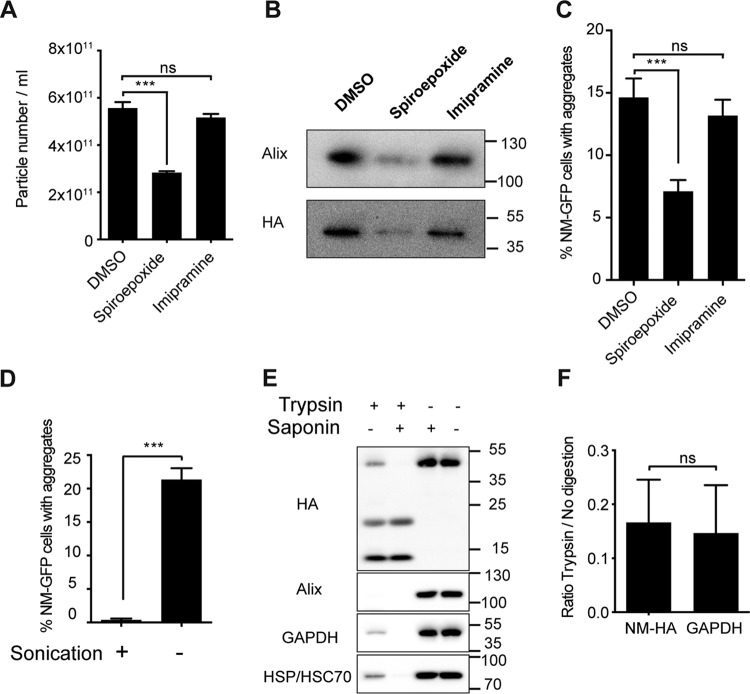
Exosomes serve as carriers of prion infectivity. (A) Determination of particle numbers. Donor clone s2E was treated with 5 µM spiroepoxide, 10 µM imipramine, or solvent control DMSO. Media were collected after 72 h, and exosome-enriched P4 fractions were isolated. The results shown are means ± SD (*n* = 3; ***, *P* < 0.001; ns, no significant difference; one-way ANOVA). (B) Samples were subjected to Western blot analysis to determine the levels of Alix and NM-HA. The values to the right are molecular sizes in kilodaltons. (C) Samples were analyzed for aggregate-inducing activity in recipient cells expressing NM-GFP^sol^. The results shown are means ± SD (*n* = 6; ***, *P* < 0.001; ns, no significant difference; one-way ANOVA). (D) Aliquots of the P4 fraction isolated from donor clone s2E were sonicated to disrupt exosomal membranes. Sonicated/nonsonicated samples were then subjected to the cell-based aggregate induction assay. The results shown are means ± SD (*n* = 6; ***, *P* < 0.0001; unpaired Student *t* test). (E) Western blot analysis of the P4 fraction (clone s2E) subjected to limited proteolysis in the presence or absence of saponin. GAPDH and Hsp70 served as markers of intraluminal proteins, and Alix is a protein associated with the exosomal membrane. (F) Fold difference in signal intensity of full-length NM-HA and GAPDH bands (with or without trypsin treatment) shown in panel E. The results shown are means ± SD (*n* = 6; ***, *P* < 0.001; ns, no significant difference; unpaired Student *t* test).

Exosomal biogenesis is regulated by sphingolipid metabolism. Decreasing the sphingolipid ceramide level with the neutral sphingomyelinase inhibitor spiroepoxide reduces exosome release, whereas imipramine, an acid sphingomyelinase inhibitor, does not (32–34). To examine the effects of these inhibitors on prion-inducing activity, exosome fractions (P4) were isolated from s2E cells treated with spiroepoxide, imipramine, or the solvent control dimethyl sulfoxide (DMSO). Treatment with spiroepoxide significantly decreased the number of vesicles in exosome fractions, while treatment with imipramine did not affect exosome secretion (Fig. 4A). Western blot analysis revealed reduced levels of the exosomal marker Alix and NM-HA in the P4 pellet of spiroepoxide-treated cells. In contrast, imipramine had no effect (Fig. 4B). Importantly, the decreased exosome release seen closely correlated with a decrease in aggregate-inducing activity (Fig. 4C).

To examine if intact exosomes are required for NM-GFP aggregate induction in recipient cells, aliquots of the exosome-enriched fraction (P4) were either left untreated or sonicated for 5 min at 100% power to disrupt exosomal membranes. Interestingly, sonication of exosomes almost eliminated their aggregate-inducing activity, arguing that intact exosomes serve as efficient carriers for prion transmission (Fig. 4D).

To assess if exosome-associated NM-HA was present outside or inside the lumen of exosomes, exosome-enriched fraction P4 derived from conditioned medium of clone s2E was subjected to limited proteolysis (Fig. 4E). Trypsin was added to degrade extraluminal proteins associated with exosomes. An aliquot of the exosome fraction was also incubated with trypsin and 0.1% saponin. Saponin permeabilizes exosomal membranes and thus allows proteolysis of luminal proteins (35, 36). Western blot analysis showed that, in the absence of saponin, the membrane protein Alix was completely degraded by trypsin, while the luminal protein glyceraldehyde 3-phosphate dehydrogenase (GAPDH) or HSP/HSC70 was at least partially protected (Fig. 4E, left). The reduction of the GAPDH and HSP70/HSC70 signals likely reflects partial disruption of exosomes even at a very low trypsin concentration and under mild digestion conditions (37). Likewise, full-length NM-HA was partially protected from trypsin in the absence of saponin. Interestingly, cleavage products of NM-HA could be detected, suggesting partial degradation by the trypsin treatment. In saponin-treated exosomes, both membrane and luminal proteins were degraded, resulting in a total loss of full-length NM-HA (Fig. 4E). The partial degradation of NM-HA and GAPDH by only trypsin treatment was quantified by calculating the ratio of full-length NM-HA or GAPDH protein bands left under trypsin treatment conditions versus a no-digestion control. No significant difference in the degradation of the two proteins was seen, suggesting that most of the NM-HA protein was localized in the lumen of exosomes secreted from s2E cells (Fig. 4F). Of note, the prion-inducing activity of trypsin-treated samples cannot be assessed, as targeting and entry of recipient cells depend on ligands on the exosome that are sensitive to proteolysis (38, 39). Because NM-HA was partially protected from proteolysis, we conclude that at least a fraction of the secreted NM-HA is present in the lumen of exosomes.

### Donor clones secrete distinct amounts of exosomes that differ in the concentration and aggregation state of NM-HA^agg^.

**FIG 5:**
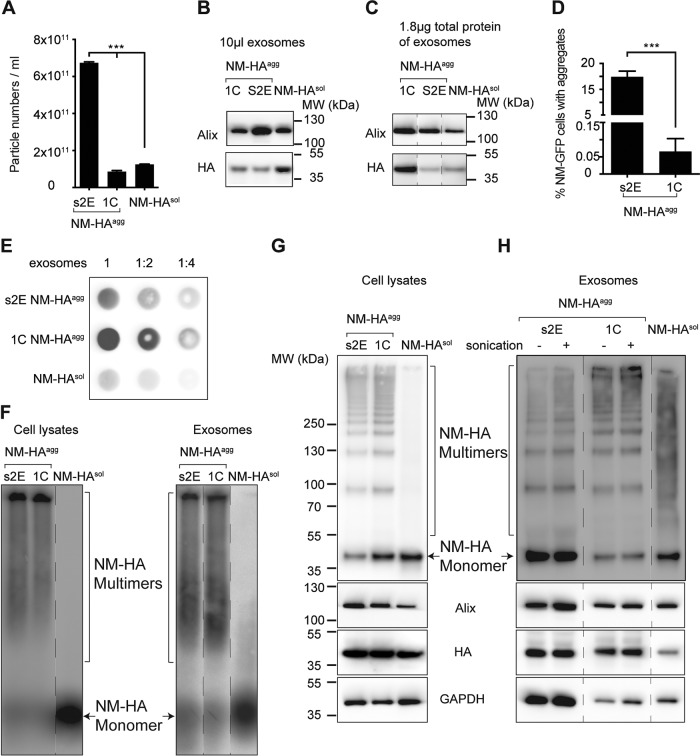
Aggregation state of NM-HA associated with exosomes. (A) Vesicle concentrations in the P4 fractions derived from media of NM-HA^agg^ clones s2E and 1C and NM-HA^sol^ cells measured by ZetaView nanoparticle tracking analysis. The results shown are means ± SD (*n* = 3; ***, *P* < 0.001; one-way ANOVA). (B, C) Western blot analyses of P4 fractions loaded at comparable volumes of isolated exosomes or adjusted to comparable total protein levels. The positions of the lanes were switched for presentation purposes (dashed lines). (D) Cell-based aggregate induction assay. Percentages of recipient cells with NM-GFP^agg^ induced by P4 exosomal fractions of donor clones 1C and s2E are shown. The results shown are means ± SD (*n* = 6; ***, *P* < 0.0001; unpaired Student *t* test). (E) Filter trap assay using P4 fractions isolated from clones s2E and 1C and NM-HA^sol^ control cells. Shown at the top are the dilutions used. (F) SDD-AGE analysis of cell lysates and exosome fractions derived from NM-HA^sol^ cells and NM-HA^agg^ clones s2E and 1C. (G, H) Glutaraldehyde cross-linking with cell lysates and exosomes from NM-HA^sol^ cells and clones s2E and 1C (top). The same amount of sample without cross-linking was also loaded as a control and analyzed for Alix, HA, and GAPDH protein levels (bottom). Extra marker lanes were removed for presentation (dashed lines).

Since secreted NM-HA from s2E cells was associated with exosomes, we wondered if the sorting of NM-HA into exosomes is an active process induced by aggregated proteins. To examine this, we determined the concentrations of extracellular particles present in P4 medium fractions from donor cells expressing NM-HA^sol^ or clones s2E and 1C with the ZetaView particle-tracking device. Significantly higher particle numbers were secreted by clone s2E than by NM-HA^sol^ cells (Fig. 5A). However, clone 1C did not show increased vesicle numbers, suggesting that NM-HA aggregation does not generally stimulate extracellular vesicle release (Fig. 5A). The increased numbers of vesicles secreted by clone s2E correlated well with the increased Alix signal detected by Western blotting (Fig. 5B). Substantial amounts of NM-HA were also associated with the exosomal fraction derived from cells that only express NM-HA^sol^, arguing that the association of NM-HA with exosomes was not dependent on the aggregation state of the protein (Fig. 5B and C). The finding that clone s2E produced significantly more exosomes than clone 1C but that the two clones did not differ drastically in the associated NM-HA levels was unexpected (Fig. 5B). Western blot analysis of similar total protein levels of exosomal samples confirmed that exosomes derived from clone 1C contained approximately six times as much NM-HA as exosomes from clone s2E (Fig. 5C). The average sizes of exosomes from 1C, s2E, and NM-HA were similar (data not shown). Notably, the exosome fraction isolated from clone s2E exhibited significantly more aggregate-inducing activity than that from clone 1C (Fig. 5D).

Our previous studies demonstrated that aggregation of cytosolic NM depends on preformed NM seeds (25). The inconsistency between NM-HA levels and aggregate induction efficiency in exosomal fractions could thus be due to different NM-HA aggregation states. To examine the aggregation state of NM-HA in exosomes, we adjusted P4 fractions isolated from the different cell lines to similar NM-HA protein levels (as assessed by Western blotting) and analyzed samples by filter trap analysis in three dilutions. SDS-resistant NM-HA aggregates were detected in exosomal pellets derived from clones 1C and s2E, while only a very faint NM-HA signal was found in the pellet derived from NM-HA^sol^ control cells (Fig. 5E). However, exosomes released from clone 1C appeared to contain more NM-HA aggregates than s2E exosomes did (Fig. 5E). Since the filter trap analysis was performed with a membrane with a 0.2-µm pore size, it is possible that only large protein aggregates were captured on the membrane. Given that exosomes from s2E are between 30 and 120 nm (Fig. 3B), it is highly likely that oligomeric species of NM-HA or aggregates smaller than 0.2 µm were packaged in exosomes. Semidenaturing detergent-agarose gel electrophoresis (SDD-AGE) (40) demonstrated the presence of polymeric NM-HA in cell lysate and exosome fractions of clones s2E and 1C but was unable to reveal major differences in the aggregation state of NM-HA derived from the two clones (Fig. 5F). Given that both filter trap analysis and SDD-AGE detect SDS-resistant protein aggregates, a cross-linking agent was used to detect non-SDS-resistant oligomeric NM-HA. The cross-linking agent glutaraldehyde has been used successfully to preserve the assembled state of the putative α-synuclein oligomers (41, 42). The cross-linking protocol was adapted for NM-HA protein in cell lysates and exosome samples from NM-HA^sol^, s2E, and 1C cells. Comparable total protein of lysates from cells was subjected to cross-linking and analyzed by SDS-PAGE. Exosome fractions were adjusted to comparable NM-HA protein concentrations before cross-linking (Fig. 5H, bottom). Multimerization of NM-HA was observed in cell lysates and exosome fractions of clones 1C and s2E (Fig. 5G and H, top). NM-HA multimerization was not a biochemical artifact of cross-linking, as no multimers were observed in control NM-HA^sol^ cells (Fig. 5G). Strikingly, the patterns of cross-linked NM-HA multimers in exosomes from clones s2E and 1C differed (Fig. 5H). Exosome fractions derived from clone s2E contained significantly more soluble than highly aggregated NM-HA. Distinct smaller-size oligomers were highly abundant. In contrast, exosome fractions from clone 1C were enriched in higher-order multimers and contained less small oligomers and monomers. Sonication of exosomal fractions prior to glutaraldehyde cross-linking did not destroy the specific banding pattern. Only diffuse polymeric NM-HA bands were detected in exosomes from NM-HA^sol^ (Fig. 5H). As NM-HA^sol^ in exosome preparations from 1C or s2E was not SDS resistant (Fig. 5F), this diffuse staining might be due to cross-linking of soluble NM-HA in tightly packed exosomes. Given that similar distributions of NM-HA multimers were detected in s2E and 1C cell lysates (Fig. 5G), it is tempting to speculate that the sorting of protein aggregates into exosomes is a selective process that controls the amount and aggregation state of sorted proteins. Altogether, our data strongly suggest that the high vesicle numbers associated with lower-order NM-HA multimers are most efficient in aggregate cell-cell transmission and induction in recipient cells.

## DISCUSSION

In this study, we demonstrate that mouse neuroblastoma cells transmit cytosolic prions derived from the yeast Sup35 NM prion domain not only by direct cell contact (26) but also by secreting prion infectivity into the extracellular space in association with membrane-bound vesicles. The presence of the flotillin, Alix-1, and Tsg101 exosome marker proteins in NM-HA-containing, infectious OptiPrep fractions and the cup-shaped appearance of the extracellular vesicles argue that these vesicles represent exosomes. The partial protection of NM-HA from tryptic proteolysis strongly suggests that at least a fraction of the NM-HA is luminal. Exosomes served as carriers of NM-HA aggregates for internalization by recipient cells and were capable of inducing the aggregation of cytosolically expressed soluble NM protein. Induced NM aggregates in recipient cells behaved as prions, continuously seeding aggregation of ectopically expressed NM. Cell clone s2E proved to be particularly efficient at inducing the prion state in bystander cells. Comparison of exosome secretion and NM-HA aggregation revealed that both clones released considerable amounts of aggregated NM-HA, with clone s2E producing significantly more exosomes. Strikingly, in exosome fractions derived from clone s2E, low-molecular-weight oligomers composed of two to five monomers appeared highly abundant. In contrast, exosome fractions from less efficient donor clone 1C were enriched in higher-order multimers.

Cytosolic NM prion induction by direct cell-cell contact depends on the transfer of NM aggregates that seed the aggregation of soluble NM in the recipient cell (26, 27). The exact size of the biologically active NM-HA seed is unknown. In direct coculture experiments, we have previously demonstrated coaggregation of induced NM-GFP aggregates in recipient NM-GFP^sol^ cells with internalized HA-tagged NM from donor NM-HA^agg^ cells (26, 27). Of note, coaggregation of microscopically visible donor and recipient NM was a rare event during direct cell contact (26, 27), indicating that smaller oligomers might also act as seeds. So far, we did not observe any coaggregation of internalized NM-HA seeds and induced NM-GFP aggregates after the addition of exosomes to recipient cells. The cross-linking analysis of NM-HA proteins in cells lysates and exosome preparations from clone s2E and 1C cells revealed similar NM-HA aggregation patterns in cell lysates but distinct patterns in exosomes, with lower-order multimers secreted by clone s2E and more abundant higher-order polymers secreted by clone 1C. These results may explain our inability to observe coaggregation of donor and recipient NM, as biologically active, seeding-competent NM polymers might be too small to be visualized by conventional confocal microscopy. It is unclear how internalized NM polymers are released into the cytosol for recruitment and seeding of endogenous NM. It is possible that NM-containing vesicles fuse with the limiting membrane of an endocytic compartment or directly rupture membranes to gain access to the cytosol (43–45).

The finding that NM prions coopt the exosome biogenesis pathway for intercellular spreading and infection is consistent with the horizontal-transmission behavior of mammalian prions derived from precursor protein PrP. Both PrP^C^ and the disease-associated, infectious isoform PrP^Sc^ have been detected in exosomal fractions isolated from cultured cells and body fluids (5, 6, 31, 46–48). Importantly, prions are not the only protein aggregates that exploit the intercellular vesicle trafficking pathway for intercellular dissemination. Over the last decade, exosomes have been shown to serve as carriers of other disease-related protein aggregates, such as Aβ and Tau (49–52) in Alzheimer’s disease, α-synuclein in Parkinson’s disease (35, 53, 54), and Cu, Zn superoxide dismutase (SOD1) in a cellular model of amyotrophic lateral sclerosis (ALS) (55–57). Thus, exosomes might generally facilitate the exchange of protein particles between cells.

Clustering and aggregation have been proposed as a trigger to reroute proteins into exosomes (58, 59) in order to lower the pathogenic protein particle burden (44). However, in agreement with the sorting of both soluble and aggregated Sup35 into extracellular vesicles by yeast (23), we found that both NM-HA isoforms were packaged into secreted vesicles. Thus, higher-order oligomerization does not appear to be required for vesicle-mediated secretion of Sup35 or its derivatives. Interestingly, in a *Caenorhabditis elegans* prion model, cell-to-cell spreading of Sup35 NM has been shown to occur via lysosomal vesicles in an autophagy-dependent manner (60). So far, it is unclear if the encapsulation of Sup35 NM into secreted vesicles is a stochastic or selective process. As NM shows little sequence homology with mammalian proteins, sorting into secreted vesicles is unlikely to depend on a cargo-specific sequence motif that mediates interaction with sorting machineries.

The formation of protein aggregates with a cross-beta structure has long been considered a pathological hallmark of so-called amyloid diseases. However, growing evidence argues that some amyloids fulfill biological functions even in mammals (61, 62). In lower eukaryotes, heritable amyloids with self-replicating properties can provide an adaptive advantage in lethal environments (17). Curiously, algorithms designed to identify prion-like domains predict that approximately 1% of human proteins contain domains with compositional similarity to yeast prion domains (14, 63). A growing number of these proteins have been shown to form cytoplasmic inclusions that are associated with neurodegenerative diseases (64). The fused in sarcoma (FUS) protein, the TAR DNA-binding protein of 43 kDa, hnRNPA1, and hnRNPA2/B1 form intracellular inclusions in frontotemporal lobe degeneration or ALS. Prion-like domains are usually present in low-complexity domains and enriched in asparagine and glutamine residues. Such domains can mediate liquid phase separation, resulting in the assembly of membraneless compartments such as stress granules (65). The exact molecular mechanism of granule formation and the underlying changes in protein structure are poorly understood (66–68). Recent studies argue that liquid phase separation and fibrillization are distinct processes. Still, the ability of certain proteins to mediate liquid phase separation might increase their aberrant fibrillization because of a high local concentration (69).

Interestingly, some human proteins with prion-like domains also have confirmed seeding activity in the disease-associated state, suggesting that they could also be intercellularly disseminated (64). A comparison of a list of 49 human RNA-binding proteins harboring prion-like domains (24) with the ExoCarta exosome protein, RNA, and lipid database (70) (http://www.exocarta.org) reveals that 71% of those proteins have been identified in exosomal fractions. Further research is required to understand whether the dissemination of soluble or proteinaceous particles composed of proteins with prion-like domains is generally associated with disease. As proteins with prion-like domains normally form functional protein assemblies in response to environmental changes, it is tempting to speculate that the intercellular transfer of those assemblies is involved in physiological phenotype regulation.

## MATERIALS AND METHODS

### Cell cultures.

N2a cells were cultured in Opti-MEM (Gibco) supplemented with glutamine, 10% (vol/vol) fetal bovine serum (FBS; PAN-Biotech GmbH), and antibiotics. All cells were incubated at 37°C in 5% CO_2_. N2a cell clones 2E and 1C stably propagating NM-HA aggregates (N2a NM-HA^agg^) and N2a cells stably producing soluble NM-HA (N2a NM-HA^sol^) or NM-GFP (N2a NM-GFP^sol^) have been described previously (25, 26). All experiments were performed with cells that were passaged less than 20 times past defrosting. The total numbers of viable cells and the viability of cells were determined with the Vi-CELLXR Cell Viability Analyzer (Beckman Coulter). To prepare exosome-depleted medium, FBS was ultracentrifuged at 100,000 × *g* and 4°C for 20 h. Medium supplemented with the exosome-depleted FBS and antibiotics was subsequently filtered through a 0.22- and a 0.1-µm filter sterilization device (Millipore).

### Induction of NM-GFP aggregation by transfer of conditioned medium.

NM-HA^agg^ clone s2E was plated into T175 flasks at different cell densities. After 3 days of culture, supernatants were harvested. Residual cells and cell debris were pelleted by three-step differential centrifugation at 300 × *g* for 10 min, 2,000 × *g* for 20 min, and 16,000 × *g* for 30 min. The recipient NM-GFP^sol^ cells were plated onto 96-well plates for 1 h and subsequently exposed to the conditioned medium for 24 h. Cells were fixed with 4% paraformaldehyde, and nuclei were stained with Hoechst.

### Aggregate induction assay.

Recipient NM-GFP^sol^ cells were cultured on a CellCarrier-384 black microplate (PerkinElmer) at 10^4^/well for 2 h and then treated with 5 to 10 µl of prepared samples (isolated exosomes, insoluble protein extract, or recombinant NM fibrils). After an additional 16 h, cells were fixed in 4% paraformaldehyde and nuclei were counterstained with Hoechst. Cells were imaged with a CellVoyager CV6000 automated confocal microscope (Yokogawa Inc.) with a 20× objective. Maximum-intensity projections were generated from Z-stacks. Images of 16 fields per well were taken. On average, a total of 3 × 10^4^ to 4 × 10^4^ cells per well and at least three wells per treatment were analyzed.

### Exosome isolation.

Exosomes were prepared from N2a NM-HA^agg^ or NM-HA^sol^ cells 17 passages postthawing. Briefly, 1.8 × 10^6^ cells were seeded into a T175 flask in 35 ml of exosome-depleted medium. Cell culture supernatants were collected after 3 days. At this time point, cells had proliferated to approximately 5 × 10^7^ viable cells per T175 flask with 98% viability, on average. Cells and cell debris were pelleted by differential centrifugation (300 × *g*, 10 min; 2,000 × *g*, 20 min; 16,000 × *g*, 30 min). The remaining supernatant was subjected to ultracentrifugation (UC; 100,000 × *g*, 1 h) with a 45Ti or SW32Ti rotor (Beckman Coulter). The pellet was rinsed with PBS and ultracentrifuged again with an SW55Ti rotor (100,000 × *g*, 1 h).

### OptiPrep density gradient.

For the discontinuous iodixanol gradient, a slightly modified form of a previously published protocol was used (71, 72). Briefly, 8.3 ml of the 60% (wt/vol) stock OptiPrep solution (Sigma) was mixed with 1 ml of 10× PBS and 0.7 ml of Milli-Q water to a final concentration of 50%. This solution was diluted to final OptiPrep concentrations of 40, 20, 10, and 5% in 1× PBS. The gradient was formed by carefully layering 3 ml of the 40, 20, and 10% solutions and 2.5 ml of the 5% solution in 14- by 89-mm polyallomer tubes (Beckman Coulter). For OptiPrep density gradient isolation of exosomes, the 100,000 × *g* pellet from 630 ml of culture supernatant (18 T175 flasks) was resuspended in 500 µl of PBS and overlaid on the gradient. The gradient was subjected to high-speed centrifugation at 100,000 × *g* for 19 h at 4°C with an SW41Ti rotor (Beckman Coulter). Twelve fractions of 1 ml each were collected from the top of the gradient, diluted with PBS in 5 ml, and centrifuged at 100,000 × *g* for 1 h at 4°C (SW55Ti rotor; Beckman Coulter). The pelleted fractions were resuspended in 100 µl of PBS and then used for further experiments. As a control, 500 µl (100 µM, monomer equivalent) of *in vitro*-formed recombinant Sup35 NM fibrils or 500 µl of insoluble pellet isolated from s2E cell lysate from two T175 flasks were loaded onto the OptiPrep gradient. The insoluble pellet was separated from soluble proteins via centrifugation at 20,000 × *g* for 20 min. To determine the density of each fraction, a negative-control gradient with 500 µl of PBS instead of samples was performed in parallel. Fractions were diluted 1:4 with water, and the absorbance at 340 nm was measured in an F-bottom 96-well PS plate (Brand) with a microplate reader (BMG Labtech). The density of the iodixanol solutions was calculated according to Table 3 of application sheet C52 (Axis-Shield PoC).

### Determination of extracellular vesicle size and number.

A ZetaView PMX 110-SZ-488 Nano Particle Tracking Analyzer (Particle Metrix GmbH) was used to determine the size and number of isolated extracellular vesicles. The instrument captures the movement of extracellular particles by utilizing a laser scattering microscope combined with a video camera. For each measurement, the video data are calculated by the instrument, showing the velocity and size distribution of the particles. For nanoparticle tracking analysis, the Brownian motion of the vesicles from each sample was monitored at 22°C with properly adjusted equal shutter and gain. At least six individual measurements of 11 subvolumes (positions) within the measurement cell and around 2,200 traced particles in each measurement were detected for each sample.

### Drug treatment.

Treatment of NM-HA^agg^ s2E cells with spiroepoxide (5 µM; Santa Cruz), imipramine (10 µM; Sigma), or DMSO was performed for 72 h in exosome-depleted medium in T175 flasks. Afterward, the total numbers of viable cells and their viability upon drug treatment were determined with the Vi-CELLXR Cell Viability Analyzer (Beckman Coulter). Exosomes were isolated from the conditioned medium via UC and processed for the aggregate induction assay as described above.

### EM.

EM imaging of exosome preparations was performed as previously described (29). Briefly, 100,000 × *g* pellets were fixed in 2% paraformaldehyde; loaded onto glow-discharged, Formvar-carbon-coated EM grids (Plano GmbH); contrasted in uranyl-oxalate (pH 7) for 5 min; and embedded in uranyl-methyl cellulose for 5 min. Samples were examined with a JEOL JEM-2200FS transmission electron microscope at 200 kV (JEOL).

### Filter trap assay and Western blotting.

The 100,000 × *g* pellets from N2a NM-HA^sol^ and N2a NM-HA^agg^ clones s2E and 1C (adjusted to similar amounts of NM-HA protein, 1:2 and 1:4 dilutions) were resuspended in 2% SDS and loaded onto a preequilibrated nitrocellulose membrane (0.2-µm pore size; Invitrogen) with a Whatman Minifold I dot blotting apparatus (GE Healthcare). Wells were rinsed five times with 200 µl of filter trap assay buffer (1% SDS and 50 mM EDTA in PBS). The membrane was incubated overnight at 4°C with anti-HA 3F10 antibody (1:1,000; Roche) after blocking in 5% skimmed milk for 1 h. For Western blot analysis, protein concentrations were measured by Quick Start Bradford protein assay (Bio-Rad) and proteins were separated on NuPAGENovex 4 to 12% bis-Tris protein gels (Life Technologies) and then transferred onto a polyvinylidene difluoride membrane (GE Healthcare) in a wet blotting chamber. Western blot analysis was performed with mouse anti-Alix (1:1,000; BD Bioscience), rabbit anti-flotillin rbt 3253 (1:1,000; NEB), rabbit anti-Tsg101 ab30871 (1:500; Abcam), rat anti-HA 3F10 (1:1,000; Roche), rabbit anti-calnexin C4731 (1:1,000; Sigma), mouse anti-GAPDH 6C5 (1:5,000; Abcam), and mouse anti-Hsc/Hsp70 N27F3-4 (1:1,000; ENZO) antibodies. The membrane was incubated with Pierce ECL Western blotting substrate (ThermoFisher Scientific) according to the manufacturer’s recommendations.

### Trypsin treatment with or without saponin.

Aliquots of exosome-enriched fraction P4 were treated with 0.05% trypsin in the presence or absence of 0.1% saponin for 1 min at 37°C. Negative controls not treated with saponin and/or trypsin were included. Proteolysis was terminated immediately at 96°C for 10 min.

### Glutaraldehyde cross-linking.

Ten-microliter volumes of cell lysate (10 µg) or exosomal sample (adjusted to similar amounts of NM-HA proteins) from NM-HA^sol^, s2E, and 1C were incubated with 5 µl of glutaraldehyde (final concentration, 0.005%) for 15 min at 37°C. The reactions were stopped by the addition of 1 µl of 1 M Tris-HCl (pH 8). NM-HA multimers were separated via SDS-PAGE and detected by Western blotting as described before.

### SDD-AGE.

SDD-AGE was performed as previously described (40). Briefly, 10 µg of cell lysates or exosome fractions with similar NM-HA contents were incubated with 3× sample buffer containing 8% SDS for 5 min at room temperature. SDS-resistant proteins were separated on an agarose gel with 0.1% SDS and transferred to a membrane. NM-HA was detected in accordance with the standard Western blotting protocol.

### Image analysis.

Image analysis was performed with the CellVoyager Analysis support software. An image analysis routine was developed for single-cell segmentation and aggregate identification (Yokogawa Inc.). The total number of cells was determined on the basis of the Hoechst signal, and recipient cells were detected by their GFP signal. Green aggregates were identified via morphology and intensity characteristics. The percentage of recipient cells with NM-GFP^agg^ was calculated as the number of aggregate-positive cells per total recipient cells set to 100%. A small percentage (<1%) of false-positive induced recipient cells was detected because of the heterogeneity in GFP expression of individual cells. The mean percentage of false positives determined in control NM-GFP^sol^ cells was subtracted from all of the samples. Of note, negative values were sometimes obtained when no induction was observed. For data presentation, the minimum range of the *y* axis was set to 0.

### Immunofluorescence staining and confocal microscopy analysis.

Cells were fixed in 4% paraformaldehyde and permeabilized in 0.5% Triton X-100. For antibody staining, cells were incubated for 2 h at room temperature with Alexa Fluor 647-conjugated mouse anti-HA TANA2 antibody (1:500; MBL) in 5% (vol/vol) ChemiBLOCKER (Millipore). Nuclei were counterstained for 15 min with 4 µg/ml Hoechst 33342 (Molecular Probes). Ninety-six- and 384-well plates were scanned with CellVoyager CV6000 (Yokogawa Inc.). Confocal laser scanning microscopy was performed with a Zeiss LSM 700 laser-scanning microscope (Carl Zeiss). Maximum-intensity projections were generated from Z-stacks.

### Statistical analysis.

All statistical analyses were performed with Prism 6.0 (GraphPad Software). Statistical intergroup comparisons were performed by one-way analysis of variance (ANOVA) with a Bonferroni posttest or Student *t* test. *P* values of <0.05 were considered significant. All experiments were performed in triplicate or sextuplicate and repeated at least two times. Error bars represent the standard deviations (SD).

## SUPPLEMENTAL MATERIAL

Figure S1  Automated image analysis for aggregate induction assay. Shown are confocal images from CellVoyager CV6000 and automated image analysis for green cell segmentation (A), identification of nuclei (B), and detection of induced green cells (including aggregates) (C). (A) Images of NM-GFP^sol^ recipient cells as a negative control. (B, C) Images of recipient cells induced for 16 h with exosomes from N2a NM-HA^agg^ s2E. Nuclei were stained with Hoechst (blue). Scale bar, 5 µm. Download Figure S1, TIF file, 1 MB

Figure S2  Fractionation of insoluble proteins in s2E cell lysate by OptiPrep density gradient centrifugation. Confluent s2E cells were lysed, and the lysate was cleared of cell debris by low-speed centrifugation. Insoluble NM-HA was subsequently pelleted (20,000 × *g* for 20 min), and proteins in the pellet fraction were separated with an OptiPrep density gradient. The different fractions were analyzed for aggregate-inducing activity in recipient NM-GFP^sol^ cells (means ± SD; *n* = 3) and for the distribution of Alix, Flotilin-1, and NM-HA via Western blotting. The aggregate induction rates correlated with the signal of NM-HA proteins, broadly distributed in fractions 1 to 10. Download Figure S2, TIF file, 1.4 MB

Movie S1  Live-cell imaging of NM-GFP aggregate induction by exosome-enriched P4 fractions derived from donor NM-HA^agg^ clone s2E. Recipient cells expressing NM-GFP^sol^ were exposed to exosome-enriched fraction P4, and live-cell imaging was performed 0 to 16 h postexposure with 6-min intervals between frames. Note that induced aggregates are transmitted to both daughter cells during cell division. Download Movie S1, MOV file, 4 MB
