# Supplementary figures and images for: Horizontal Transmission of Cytosolic Sup35 Prions by Extracellular Vesicles
Source: mBio. 2016 Jul 12;7(4):e00915-16. doi: 10.1128/mBio.00915-16 (PMC4958257; doi:10.1128/mBio.00915-16)

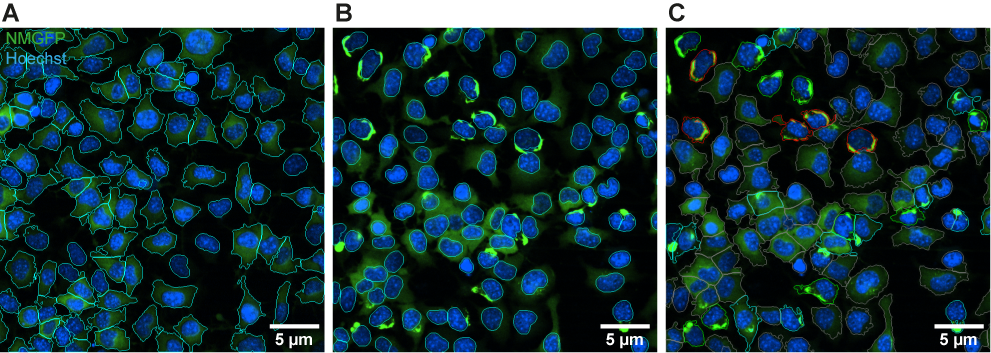

Supplement: Figure S1 — Automated image analysis for aggregate induction assay. Shown are confocal images from CellVoyager CV6000 and automated image analysis for green cell segmentation (A), identification of nuclei (B), and detection of induced green cells (including aggregates) (C). (A) Images of NM-GFPsol recipient cells as a negative control. (B, C) Images of recipient cells induced for 16 h with exosomes from N2a NM-HAagg s2E. Nuclei were stained with Hoechst (blue). Scale bar, 5 µm. Download [file mbo004162894sf1.tif]

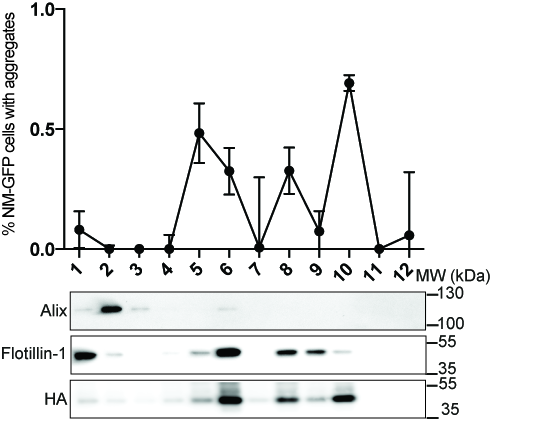

Supplement: Figure S2 — Fractionation of insoluble proteins in s2E cell lysate by OptiPrep density gradient centrifugation. Confluent s2E cells were lysed, and the lysate was cleared of cell debris by low-speed centrifugation. Insoluble NM-HA was subsequently pelleted (20,000 × g for 20 min), and proteins in the pellet fraction were separated with an OptiPrep density gradient. The different fractions were analyzed for aggregate-inducing activity in recipient NM-GFPsol cells (means ± SD; n = 3) and for the distribution of Alix, Flotilin-1, and NM-HA via Western blotting. The aggregate induction rates correlated with the signal of NM-HA proteins, broadly distributed in fractions 1 to 10. Download [file mbo004162894sf2.tif]
